# Aging has occurred rapidly in the facial fracture population – are we ready?

**DOI:** 10.1007/s00784-025-06640-7

**Published:** 2025-11-12

**Authors:** Elias Kultanen, Aleksi Haapanen, Roope Mustasilta, Tero Puolakkainen, Anne Abio, Hanna Thorén, Johanna Snäll

**Affiliations:** 1 Department of Oral and Maxillofacial Diseases, University of Helsinki and Helsinki University Hospital, Helsinki, Finland; 2 Department of Oral and Maxillofacial Diseases, Central Finland Central Hospital Nova, Jyväskylä, Finland; 3 Research Center for Child Psychiatry, University of Turku, Turku, Finland; 4 INVEST Research Flagship Center, University of Turku, Turku, Finland; 5 Department of Oral and Maxillofacial Surgery, Department of Oral and Maxillofacial Diseases, University of Turku, Turku University Hospital, Turku, Finland

**Keywords:** Aging, Facial injuries, Facial fractures, Epidemiology, Falls, Elderly

## Abstract

**Objectives:**

It is well known that the global population is aging. The aim of this study was to compare the annual changes in age and sex profile among patients with facial fractures over an 8-year period.

**Materials and methods:**

This retrospective cohort study included facial fracture patients treated at a tertiary trauma center between January 2013 and October 2020. The study examined age- and sex-related changes over time, and investigated the association between aging, ground-level falls, and the need for surgical treatment.

**Results:**

In total, data was collected for 4170 facial fracture patients included in the study. Of these patients, 2957 (70.9%) were male. The mean age of the study population was 45.7 years old, with a median age of 42.4 years. The most common mechanism of injury was ground-level falls, accounting for 31.8% of cases, followed by assaults at 27.6%, and traffic collisions at 20.1%. The increase in mean age over time was statistically significant both for females (*p* = 0.028) and males (*p* = 0.001). The incidence of ground-level falls among males showed a statistically significant increase over time (*p* < 0.001).

**Conclusions:**

This study provides insight into the ongoing change in the characteristics of patients suffering from facial fractures. Overall, clinicians will encounter increasingly more elderly male patients with facial fractures due to ground-level falls.

**Clinical relevance:**

We encourage units treating facial fractures to consider the care processes for the elderly, as their proportion within patient populations continues to increase.

## Introduction

It is well known that fertility rates are declining, and life expectancy is increasing, particularly in Western countries. This, in turn, has reshaped the global population age structure, with the elderly population growing rapidly [1]. The changes and effects in the age structure of the facial fracture population has not been widely studied, even though an increasing proportion of patients with facial fractures are elderly [2]. Previous studies have evaluated the impact of this change on healthcare systems, the need for surgery, treatment strategies and maxillofacial surgeons’ proficiency [3, 4], while other studies have focused on the causes of the injuries among the elderly [5, 6]. However, descriptions of aging trends across timelines and the effects on facial fracture patient populations are scarcely reported.

Elderly patients frequently require more healthcare resources, as opposed to younger patients with similar injuries [7], but the extent of this phenomenon is not sufficiently known in facial fracture patients. Due to the constantly aging population, it is increasingly important to acknowledge the developing situation and thrive to establish more effective treatment strategies for this patient group [8]. The aim of this study was to compare the annual changes in age and sex profile among patients with facial fractures over an 8-year period. The study hypothesis was that an aging trend currently exists in the facial fracture population, leading to clinically significant changes in the patient profile.

## Materials and methods

### Study design

This retrospective cohort study included facial fracture patients admitted to a tertiary trauma center (Helsinki University Hospital, Helsinki, Finland) with any type of facial fracture from January 2013 to October 2020.

## Inclusion and exclusion criteria

Data were retrieved from electronic patient records according to the ICD-code for craniofacial fracture. The data were manually reviewed, and patients with radiologically confirmed facial fractures were included in the analyses. Patients with isolated skull fractures occurring outside the facial thirds were excluded from the study.

## Study variables

The changes in patients’ age were examined over the study period, with an assessment of sex differences in aging. The association between aging and the frequency of ground-level falls, as well as the need for surgical treatment, was investigated. Age was considered as a continuous variable. Surgical treatment was defined as surgical reduction of the fracture with or without internal fixation or intermaxillary fixation.

### Ethical approval

The study protocol was approved by the Internal Review Board of the Head and Neck Centre, Helsinki University Hospital, Helsinki, Finland (HUS/356/2017 and HUS/58/2020).

### Data analysis

Percentages and frequencies for categorical variables were presented and crosstabulations were done using the Pearson Chi Square test. Means and standard deviations or median and interquartile range were presented for continuous variables. A nonparametric trend test (Jonckheere-Terpstra) was used to investigate the trend of age over time, trend of age in ground-level falls, and the mean age versus need for surgical treatment over time. The Chi Squared trend test was used to assess the change in mean age for the occurrence of ground-falls by sex. The association between the need for surgical treatment and explanatory variables was examined using logistic regression analysis. The statistical analysis was conducted using Stata 18 (StataCorp. TX, USA).

## Results

Between the years 2013 and 2020, 4170 patients suffered from facial fractures and were evaluated in the emergency units included in the present study.

Descriptive statistics of the patients are presented in Table 1. Of the 4170 patients, 2957 (70.9%) were male. The mean age of the study population was 45.7 years (median 42.4 years, range from 0.5 to 102.5 years). The most frequent mechanism of injury was fall at ground level (31.8%), followed by assault (27.6%), and traffic accidents (20.1%). The most common fracture type was exclusively midfacial fracture (46.9%). Overall, 40.2% of the patients received surgical treatment.

**Table 1:** Descriptive statistics of 4170 facial fracture patients

Variable	Number of patients	%
Total	4170	100
Sex		
Male	2957	70.9
Female	1213	29.1
Age (yr)		
Mean	45.7	
Median	42.4	
Range	0.5–102.5	
Mechanism of injury		
Fall at ground level	1328	31.8
Assault	1153	27.6
Traffic accident	839	20.1
Fall from stairs or height	429	10.3
Struck or hit by object	359	8.6
Other	62	1.5
Fracture type		
Exclusively midface	1954	46.9
Exclusively mandible	1195	28.7
Upperface	128	3.1
Combined	893	21.4
Surgical treatment		
Yes	1676	40.2
Open surgery	1405	83.8
Closed reduction*	271	16.2

*Including intermaxillary fixation

**Fig. 1:**
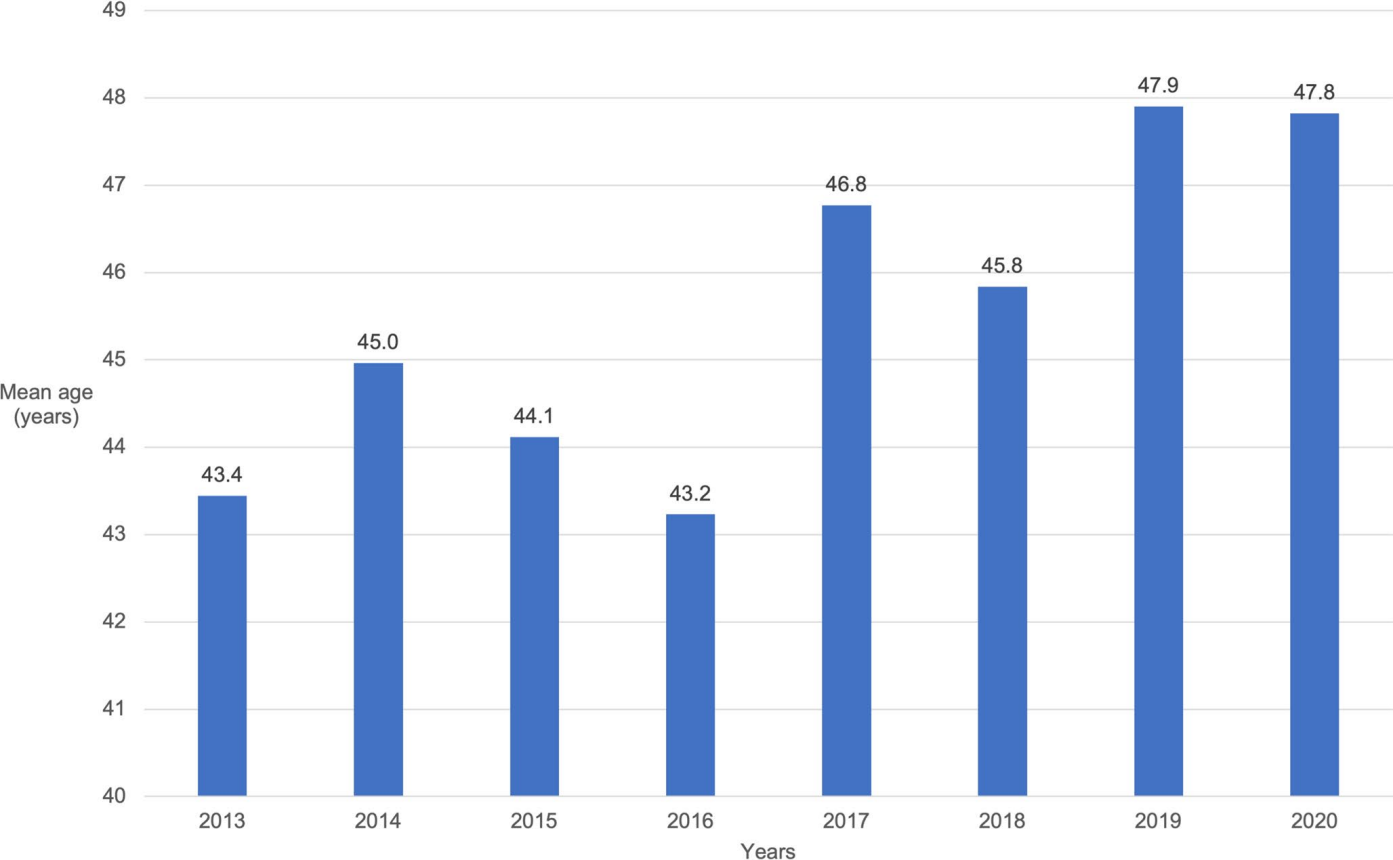
Mean age of patients included in the study over the years included in the study period (2013–2020)

**Fig. 2:**
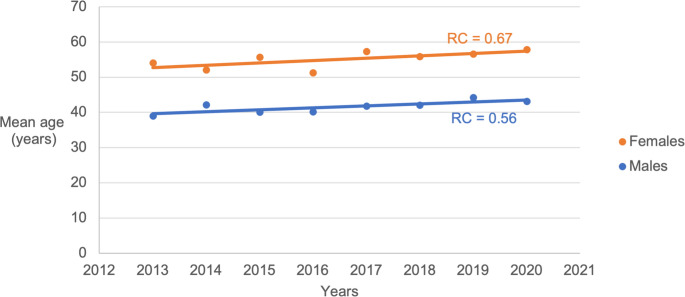
Increase in mean age among females and males over time was statistically significant for both females (*p* = 0.028) and males (*p* < 0.001). RC = regression coefficient

During the years 2013 to 2020, the patients’ mean age increased from 43.4 years old (in 2013) to 47.8 years old (in 2020) (Fig. 1). A linear regression coefficient (RC) for mean age among females (RC=0.671) and males (RC=0.558) over time was statistically significant (Fig. 2).

**Fig. 3:**
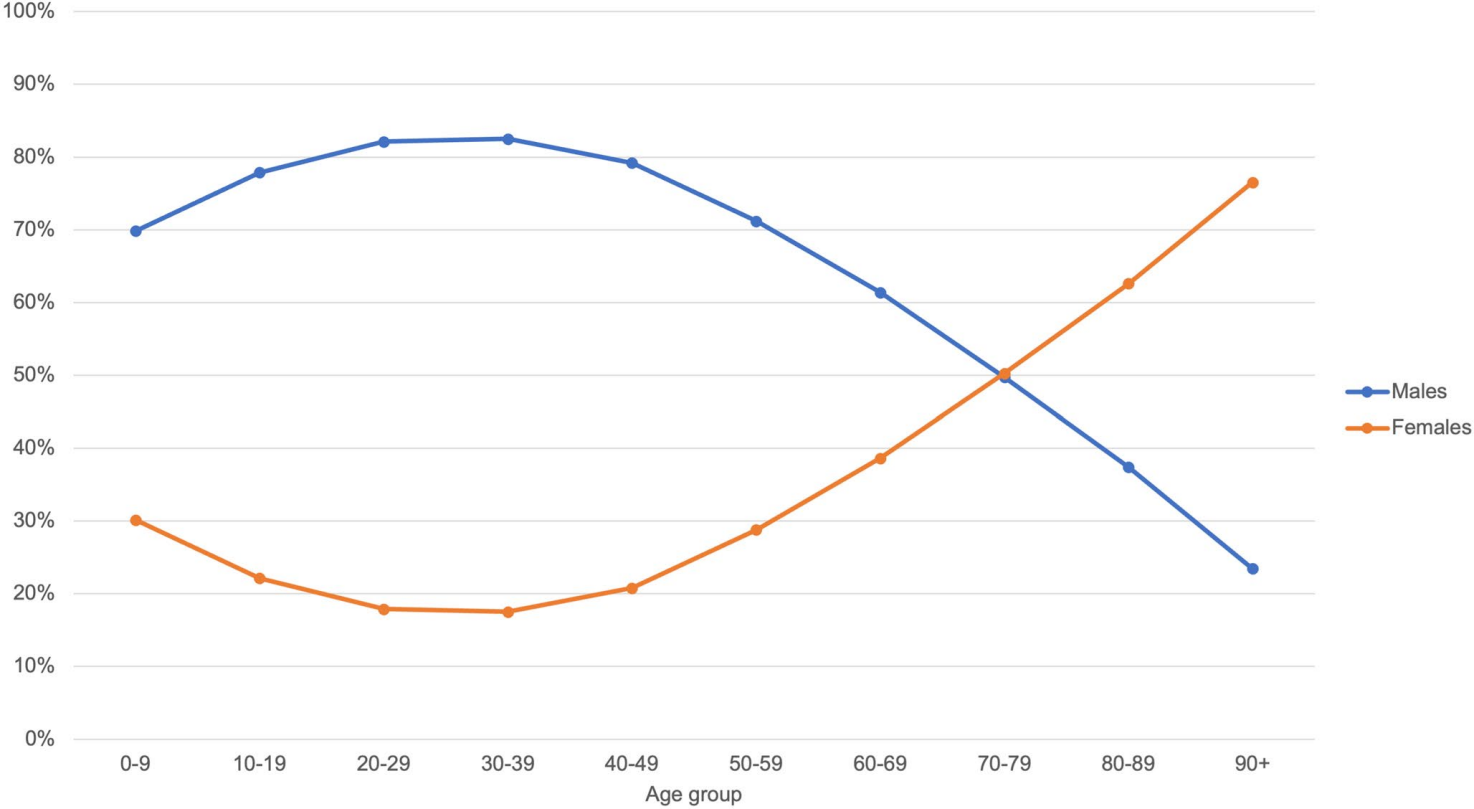
The age distribution of facial fracture patients varied by sex (males = blue, females = orange). Fractures were observed particularly in young and middle-aged male. The proportion of female increased with age, exceeding the proportion of male from the age of 80 onwards

 Significant sex-based differences were observed across age groups (Fig. 3). Among male patients, the predominant age group was 30–39 years old, accounting for 82.5% of cases, while female patients in the same age group represented 17.5%. In contrast, the most dominant age group for female patients was 90 years and over (76.5%) in relation to male patients in the same age group (23.5%). The smallest sex differences were observed in the 70–79-year-old age group, where 49.7% of patients were male and 50.3% were female.

**Fig. 4:**
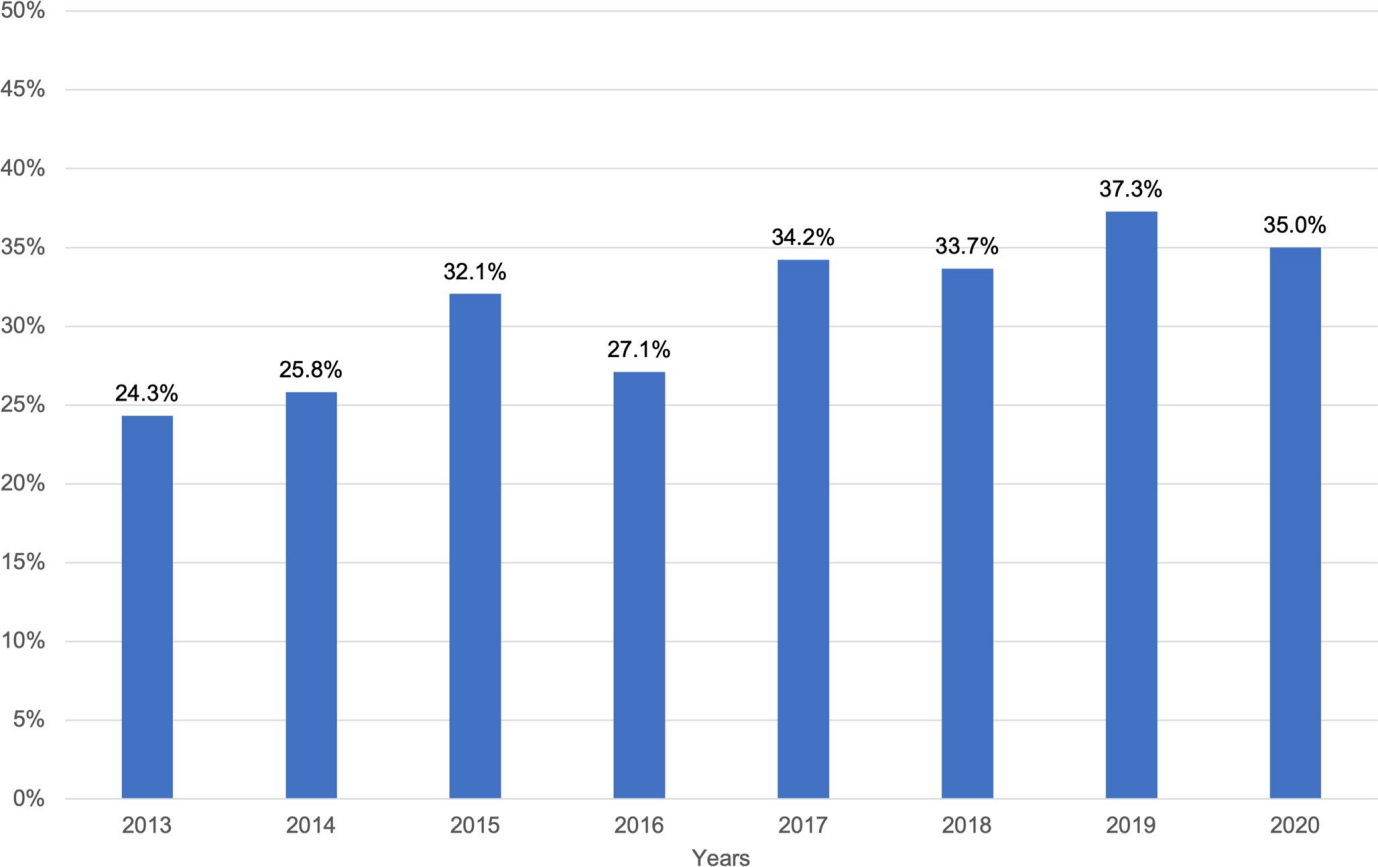
Ground level falls of all injury types (%) increased over the years included in the study period (2013–2020)

**Fig. 5:**
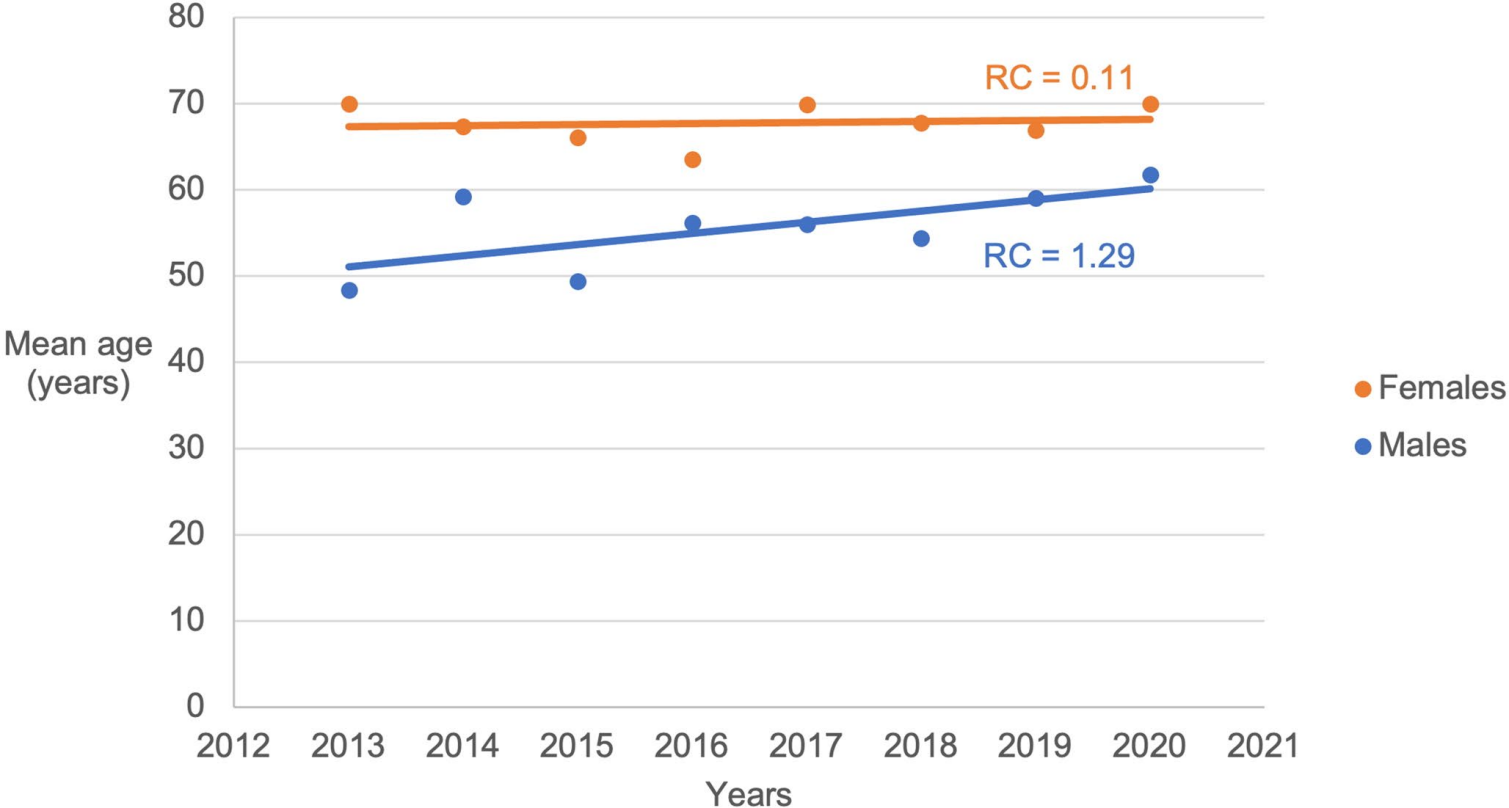
Increased trend for year and ground-level falls was statistically significant in males (*p* < 0.001) but non-significant in females *p* = 0.8202. RC = regression coefficient

 Injury mechanisms varied significantly between the studied years (p < 0.001). The proportion of ground-level falls increased from 24.3% to over one-third, with the highest incidence (37.3%) observed in 2019 (Fig. 4). Examination of ground level falls revealed a temporal change. Ground-level falls in males increased over the years (Fig. 5).

**Fig. 6:**
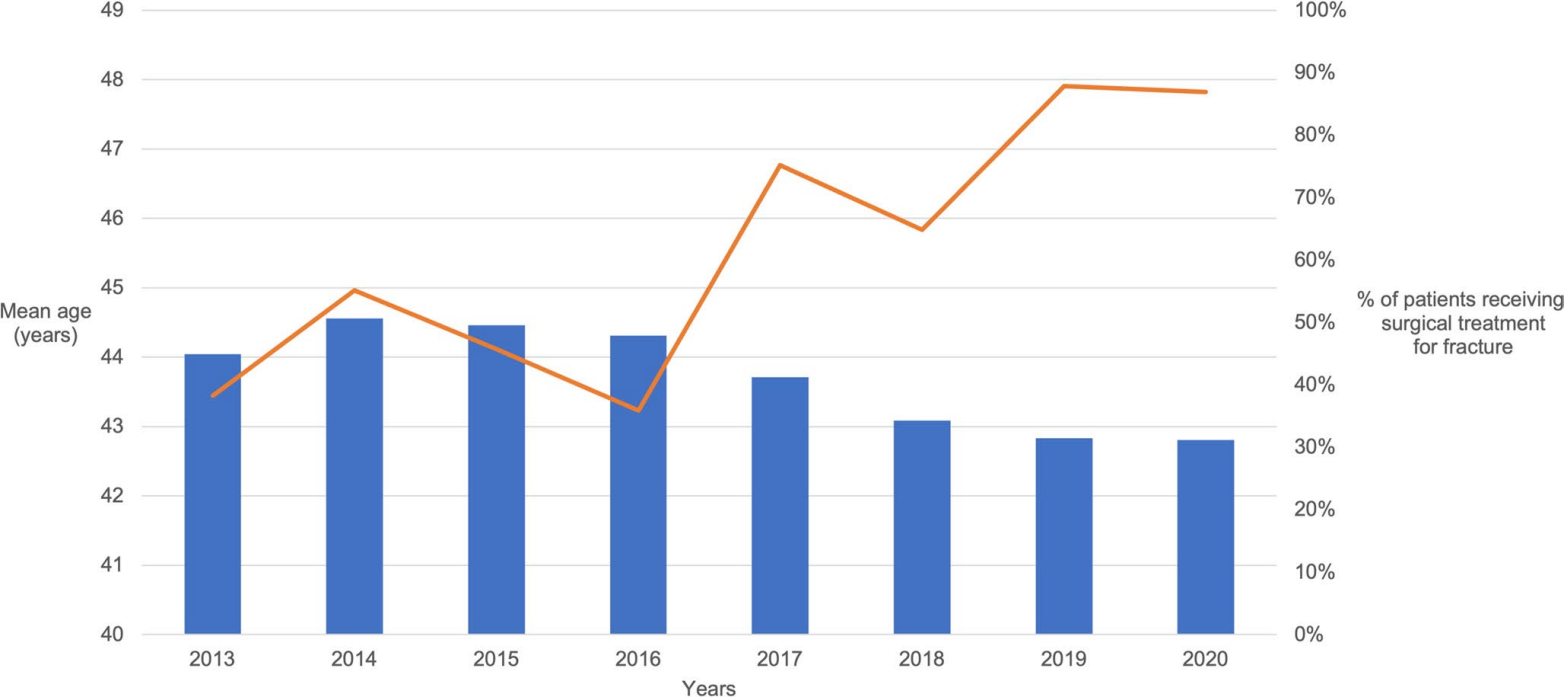
Mean age of patients (orange) increased and proportional percentage of surgical treatment (blue) decreased over the years included in the study period (2013–2020)

 Over the study period, the number of facial fractures requiring surgical treatment decreased each year (Fig. 6). In 2013, 45.0% of patients received surgical treatment for fractures, whereas in 2020, the corresponding rate was 31.2% (*p *< 0.001).

## Discussion

The global aging of the population, together with substantial progress in healthcare has led to a significant increase in the number of active older people in societies [2, 9]. The continuous aging of the global population has been noted specifically in surgical fields [1], and the present study further reinforced this trend by highlighting rapid changes in the facial fracture population (Fig. 1). Our research hypothesis was confirmed, as the effects of aging on the facial fracture population were clinically significant, even over a short period. Ground-level falls have increased, and a notable shift in the patient profile has occurred, particularly regarding the proportion of elderly men. However, aging does not appear to increase the need for surgical treatment; rather, the number of surgically treated fractures decreased during the review period. Therefore, the field of facial traumatology must adapt to aging in other ways. 

Emergency departments should first consider the unique characteristics of elderly patients. Recognizing facial injuries in this group can be challenging due to communication difficulties or other underlying health conditions. Accordingly, underdiagnosis is a recognized issue in elderly patients with facial fractures [4]. Thus, trauma assessment in the elderly requires a higher level of vigilance compared to younger patients. A specific challenge for clinicians and facial trauma units is identifying associated injuries and managing the treatment process for these injuries. For example, elderly patients frequently sustain significant injuries to other body parts, and particular attention should be given to the increased risk of traumatic brain injuries [10–14]. 

The lower need for surgical treatment observed in the elderly compared to other age groups has been reported before [8, 15]. For an individual, facial fractures may bring considerable consequences, such as functional challenges, aesthetic issues and emotional or physical stress [3]. Thus, the indications for surgery – and, most likely, in facial fracture patients’ general anesthesia – can be obvious even in the elderly, for example in dislocated open fractures of the mandible. On the other hand, the risks of general anesthesia and different treatment options must be carefully considered. Clinicians treating the elderly should be aware of possible challenges, such as the risks of general complications and the demands of the post-hospital rehabilitation process [16]. These risks and treatment options should be thoroughly evaluated, considering the patient’s quality of life and capacity for rehabilitation. 

As the patient profile continues to change, this requires that clinicians have comprehensive trauma expertise, along with structured protocols for trauma assessment and processes for inpatient care and rehabilitation, to ensure that the specific needs of elderly facial fracture patients are met. The individual consequences, coupled with the broader public health impact of facial fractures, highlight the importance of taking proactive measures, developing effective treatment strategies, and allocating resources more efficiently [17]. 

Even if all facial fractures do not require surgical treatment, the injuries can affect patients’ quality of life, at least temporarily. Restrictions to eating, vision and speaking, as well as possible associated injuries outside of the facial region can make the patient’s daily life more difficult. Even without surgical intervention, elderly patients with facial fractures often require curated and special long-term care to maintain good physical and mental health [8, 18]. Notably, the challenges are general, as these life quality considerations apply to all fields of medicine, not just facial traumatology. Thus, the significant burden of the aging population on healthcare systems must be recognized widely [3, 7, 19]. 

A notable sex difference is a specific feature in facial fracture populations. Males are known to suffer more frequently from facial injuries compared to females [3, 5, 9, 10, 17, 20–25], as was also found in our results. Males’ susceptibility specifically to injuries due to physical violence explains most of this difference [19, 26]. Our study revealed a new trend in the facial fracture population related to the increasing number of actively aging individuals, wherein these older active individuals – particularly male – are prone to falls, and consequently sustain facial fractures. This observation can be explained by the lengthening of life expectancy and the rising number of elderly men. 

Ground-level falls have been previously highlighted as the most significant injury mechanism in the elderly [2, 6, 10, 18, 23, 24, 27, 28], as was also found in the present study. In comparison, assaults, road traffic accidents and sports accidents are more common in the younger population [18, 24, 26]. Typical characteristics of the elderly predispose them to accidents in daily life. For example, elderly patients require less impact to produce injuries compared to younger people [7], and impaired motor skills, reduced perception, awareness, declined balance and eyesight make people – especially those who are older – more prone to falls [2, 29]. In addition, underlying medical conditions increase the possibility of a ground-level fall [2, 29]. 

The share of ground-level falls of all injury types showed a steady growth during the study period (from 24% in 2013 to 35% in 2020) (Fig. 4). The increasing trend is supported by previous studies [2, 6, 18, 23–25, 27, 28, 30] and was especially noticeable in the study by Boffano et al. [17]. In a European multicenter study, the results showed that in trauma centers where the mean age was higher than 40 years, the most frequent causes of maxillofacial injuries were falls. Thus, societies should invest in the prevention of falls also in terms of the prevention of facial fractures. 

The main limitation of the present study was the retrospective study design, which limited the collection of study parameters. The medical history of elderly patients would have been particularly interesting, since it would have given more specific information about the risk factors and background variables of patients who usually suffer from facial fractures. In addition, the effect of sociodemographic factors and the duration of treatment was not considered, which might have affected the results of the present study. These limitations emphasize the importance of studying this topic further. 

In conclusion, the findings of this study highlight the ongoing changes in the characteristics of patients with facial fractures. As the population continues to age, it is inevitable that clinicians will encounter an increasing number of elderly patients suffering facial fractures from ground-level falls. Oral and maxillofacial surgery teams should therefore focus on adapting evaluation strategies and developing effective treatment protocols for elderly patients with facial fractures. This trend is expected to persist, driven by the aging population, increased life expectancy, and more active lifestyles.

## Data Availability

No datasets were generated or analysed during the current study.
